# A Bacteriophage-Acquired O-Antigen Polymerase (Wzy_β_) from *P. aeruginosa* Serotype O16 Performs a Varied Mechanism Compared to Its Cognate Wzy_α_

**DOI:** 10.3389/fmicb.2016.00393

**Published:** 2016-03-31

**Authors:** Véronique L. Taylor, Jesse F. J. Hoage, Sandra Wingaard Thrane, Steven M. Huszczynski, Lars Jelsbak, Joseph S. Lam

**Affiliations:** ^1^Department of Molecular and Cellular Biology, University of GuelphGuelph, ON, Canada; ^2^Department of Systems Biology, Technical University of DenmarkKongens Lyngby, Denmark

**Keywords:** *Pseudomonas aeruginosa*, lipopolysaccharide, bacteriophage, serotype, polymerase, glycosyltransferase, O-antigen biosynthesis

## Abstract

*Pseudomonas aeruginosa* is a Gram-negative bacterium that produces highly varied lipopolysaccharide (LPS) structures. The O antigen (O-Ag) in the LPS is synthesized through the Wzx/Wzy-dependent pathway where lipid-linked O-Ag repeats are polymerized by Wzy. Horizontal-gene transfer has been associated with O-Ag diversity. The O-Ag present on the surface of serotypes O5 and O16, differ in the intra-molecular bonds, α and β, respectively; the latter arose from the action of three genes in a serotype converting unit acquired from bacteriophage D3, including a β-polymerase (Wzy_β_). To further our understanding of O-polymerases, the inner membrane (IM) topology of Wzy_β_ was determined using a dual *phoA-lacZ*α reporter system wherein random 3′ gene truncations were localized to specific loci with respect to the IM by normalized reporter activities as determined through the ratio of alkaline phosphatase activity to β-galactosidase activity. The topology of Wzy_β_ developed through this approach was shown to contain two predominant periplasmic loops, PL3 (containing an RX_10_G motif) and PL4 (having an O-Ag ligase superfamily motif), associated with inverting glycosyltransferase reaction. Through site-directed mutagenesis and complementation assays, residues Arg^254^, Arg^270^, Arg^272^, and His^300^ were found to be essential for Wzy_β_ function. Additionally, like-charge substitutions, R254K and R270K, could not complement the *wzy*_β_ knockout, highlighting the essential guanidium side group of Arg residues. The O-Ag ligase domain is conserved among heterologous Wzy proteins that produce β-linked O-Ag repeat units. Taking advantage of the recently obtained whole-genome sequence of serotype O16 a candidate promoter was identified. *Wzy*_β_ under its native promoter was integrated in the PAO1 genome, which resulted in simultaneous production of α- and β-linked O-Ag. These observations established that members of Wzy-like family consistently exhibit a dual-periplasmic loops topology, and identifies motifs that are plausible to be involved in enzymatic activities. Based on these results, the phage-derived Wzy_β_ utilizes a different reaction mechanism in the *P. aeruginosa* host to avoid self-inhibition during serotype conversion.

## Introduction

*Pseudomonas aeruginosa* is a highly successful opportunistic pathogen, due partly to its arsenal of virulence factors including, toxins, several secretion systems, and lipopolysaccharide (LPS). LPS is a complex glycolipid that comprises the majority of the outer-leaflet of the outer membrane (OM) and has three domains including lipid A, core oligosaccharide, and a distal O antigen (O-Ag). *P. aeruginosa* produces two forms of O-Ag, the homopolymeric common polysaccharide antigen (CPA), and the heteropolymeric O-specific antigen (OSA) made of a series of repeating sugar subunits (King et al., 2009). The diverse structures of OSA classify *P. aeruginosa* into 20 distinct serotypes in the International Antigen Typing Scheme (IATS) (Knirel et al., 1988; Stanislavsky and Lam, 1997).

**Figure 1:**
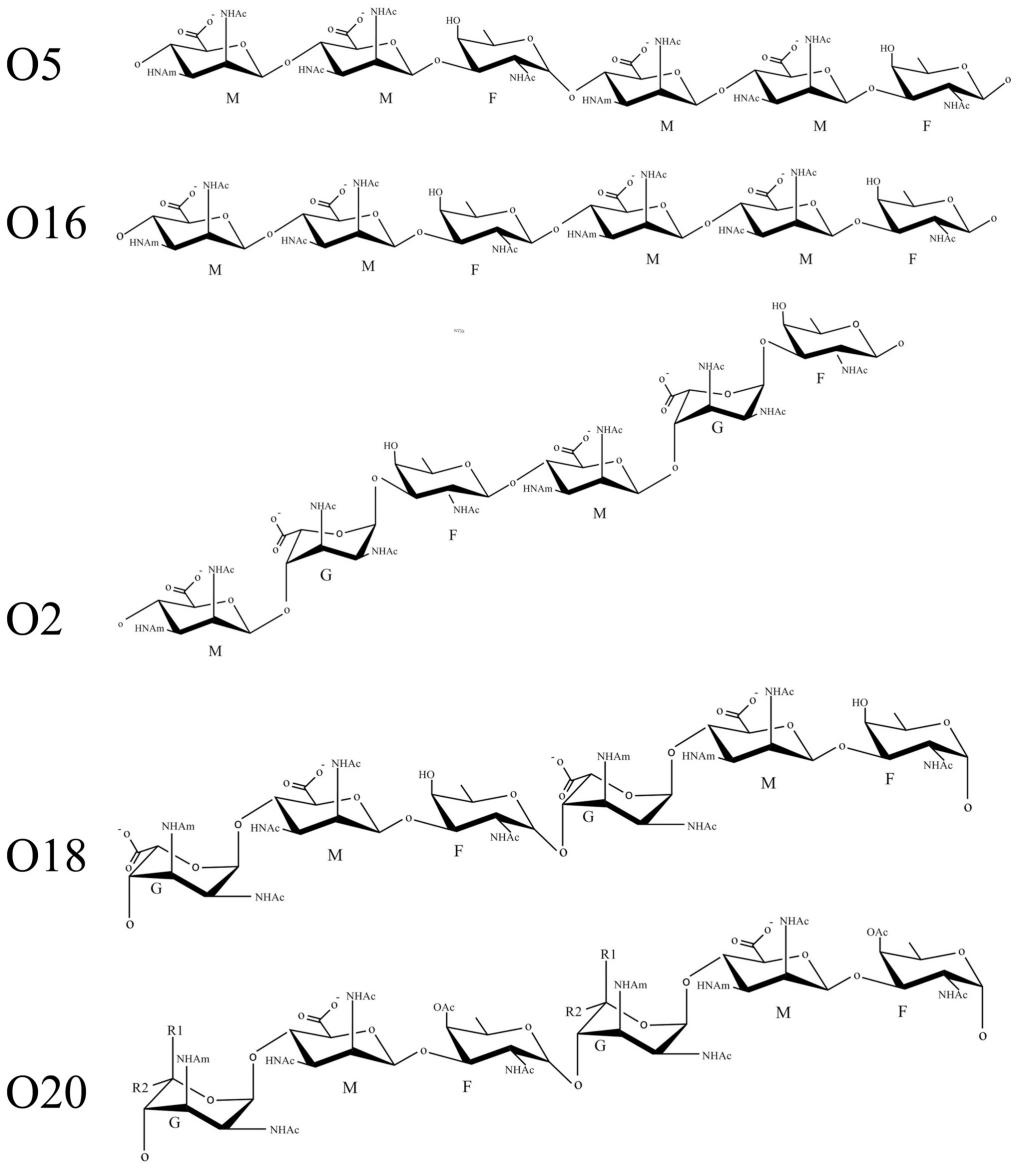
**Schematic representation of serogroup O2 sugar repeats**. The following abbreviations are used in the figure: G, guluronic acid; M, mannuronic acid; F, fucosamine; OAc, O-acetyl group; NHAc, acetamido group; NHAm, acetamidino group; R1, COOH or H; R2, H or COOH.

The OSA of *P. aeruginosa* is synthesized through the Wzx/Wzy-dependent pathway involving a series of inner membrane (IM) proteins that is highly conserved in Gram-negative and Gram-positive organisms that possess heteropolymeric glycans on the cell surface, which include O-antigen, spore coat, enterobacterial common antigen, and capsule (Islam and Lam, 2014). In this model, lipid-linked trisaccharide repeats are flipped from the cytoplasmic side to the periplasm side of the inner membrane by Wzx (O-flippase) (Islam et al., 2012), polymerized at the reducing-end by Wzy (O-polymerase) to a chain-length regulated by Wzz (polysaccharide co-polymerase, PCP) (Burrows et al., 1997). In *P. aeruginosa*, two distinct PCP proteins are encoded in the genome, named Wzz_1_ and Wzz_2_ (Daniels et al., 2002), regulating the synthesis of particular lengths of long and very-long repeats, respectively. The final step of LPS biosynthesis at the IM is ligation of the OSA to the lipid A-core by WaaL, by an inverting glycosyltransferase reaction, before the mature glycolipid is exported to the OM (Sadovskaya et al., 2000; Abeyrathne et al., 2005). The serotypes which comprise the O2 serogroup are: O2, O5, O16, O18, and O20. Although, serotype O5 and O16 of *P. aeruginosa* produce OSA with identical sugars, the structures of the two differ at the intra-molecular bond, wherein O5 is α-linked and O16 is β-linked (Figure 1).

The biosynthesis clusters of these serotypes are identical; therefore, the genes responsible for this difference must be localized elsewhere within the genome. Previously it was determined that following infection of *P. aeruginosa* strain PAO1 (serotype O5) by the D3 bacteriophage, the lysogen, AK1380, undergoes serotype conversion to serotype O16 and produces β-linked OSA (Holloway and Cooper, 1962; Kuzio and Kropinski, 1983). The D3 bacteriophage is a temperate phage of *P. aeruginosa* isolated by Holloway in 1960 (Holloway et al., 1960), which has a polyhedral head containing linear double-stranded DNA (Miller et al., 1974). Infection by the D3 phage renders the bacteria resistant to superinfection by other phages (Holloway and Cooper, 1962). The genes responsible for this phenomenon were identified in the phage genome which encodes an inhibitor of α-polymerase (Iap) and a β-polymerase (Wzy_β_) (Newton et al., 2001). The serotype converting genes are present in other *P. aeruginosa* phages: phi297 and pMG1 (Krylov et al., 2012, 2013). Further work by our group showed that in serotype O2 and O16 strains, the serotype converting unit is constitutively expressed resulting in active inhibition of the native Wzy_α_ and only the production of β-linked OSA (Kaluzny et al., 2007). Although both polymerases recognize the same substrate they share 21% sequence identity to each other (Figure S1).

Prior investigations of Wzy by several laboratories relied on validating *in silico* topology maps through quantifying enzyme activity of fusions downstream of site-targeted truncations (Daniels et al., 1998; Mazur et al., 2003; Kim et al., 2010). A caveat of *in silico* predictions is the reliance on “low energy states,” which tends to bias the localization of charged residues to more soluble environments (Elofsson and von Heijne, 2007; Bañó-Polo et al., 2012). In addition, improper localization of the N− and C− terminal ends, or the number of TMS, detected for the protein of interest will affect the orientation of subsequent loop domains (Krogh et al., 2001). Our lab determined the topology of the cognate Wzy_α_ from *P. aeruginosa* PAO1, which produces α-linked OSA, using an unbiased experimentally-derived approach based on a dual-enzyme reporter system pioneered by Alexeyev and Winkler (1999) in order to reveal previously unidentified domains. The experimental topology map revealed two novel periplasmic loops (PL) PL3 and PL5 in Wzy_α_. Intriguingly, the region of amino acid residues that make up PL3 had been predicted to localize within a transmembrane segment (TMS) in the *in silico* map. Further investigation of PL3 and PL5 determined that each of these loops contained a conserved RX_10_G tract of amino acids similar to the HX_10_G polymerase motif (Schild et al., 2005). Alanine-scanning mutagenesis of conserved Arg residues determined that their guanidinium side group, not just positive charge, is important for *in vivo* function of Wzy_α_. An earlier study has provided evidence that the guanidium side group binds carbohydrates (Dahms et al., 1993). Although the two loops possessed similar amino acid sequence, there was a drastic difference in their isoelectric point with PL3 at pI 8.59 and PL5 at pI 5.49; hence, under physiological pH, the two loops would have contrasting cationic and anionic charges, respectively. These observations and other data have led to the proposed “catch-and-release” mechanism of Wzy_α_ wherein PL3 would act to recruit the newly-flipped OSA repeat with PL5 loosely interacting with the substrate as a retaining arm (Islam et al., 2011).

In a subsequent study, an extensive alanine-scanning screen of exposed charged and polar residues was undertaken which determined that only mutated residues localized within PL3 and PL5 would lead to abrogation of the function of Wzy_α_. These observations highlighted the importance of these domains. However, two amino acids within a cytoplasmic loop of Wzy_α_, N380 and R385, when modified by site-directed mutagenesis, conferred altered chain-length modality of the LPS produced by the mutant bacteria, suggesting that these residues are involved in interactions with the co-polymerase protein, Wzz_1_ (Islam et al., 2013a). Recently the Wang group (Zhao et al., 2014) produced chemo-enzymatically derived substrates to investigate the activity of Wzy to catalyze LPS assembly *in vitro*. They showed that Wzy adopts a distributive mechanism for polymerization of O-Ag in *E. coli*, which is consistent with the catch and release model that our group had proposed earlier for Wzy proteins (Islam et al., 2011).

In this study, we determined the topology of the bacteriophage-acquired Wzy_β_ from serotype O16 using the dual-reporter system as described above. Our data allowed us to build a topology map of Wzy_β_ with 10 TMS and cytoplasmic N- and C-terminal ends, plus the existence of two large periplasmic loops designated as PL3 and PL4, wherein PL3 possesses an RX_10_G motif and PL4 contains a Wzy_C motif, a conserved domain among members of the O-Ag ligase superfamily. Both motifs were deemed essential based on evidence from alanine-scanning mutagenesis in which critical Arg and His residues were identified in the two loops, respectively. In this investigation, we demonstrate that either class of Wzy proteins possesses a dual periplasmic loops topology and PL4 in Wzy_β_ contains an essential stretch of amino acids which likely perform inverting glycosyltransferase reactions and contributing to the O16 OSA to be assembled as a β-linked polymer. This finding substantiated the existence of a possible catalytic region in polymerase proteins localized to the C-terminal region which is involved in the formation of α- or β-linked OSA structures.

## Materials and methods

### *In silico* methods

TOPCONS and HMMTOP 2.0 were used to produce a *de novo* topology map of Wzy_β_ (Bernsel et al., 2009). The *de novo* map was generated using the web-based Protter software (Omasits et al., 2014). To generate the experimentally-derived topology map, once the truncations of *wzy*_β_ were localized, the designation was inputted using the Advanced setting of HMMTOP 2.0 (Tusnády and Simon, 1998).

### Generating the truncation library upstream of the dual-reporter

The *wzy*_β_ sequence was amplified from a previously prepared plasmid construct (Kaluzny et al., 2007) and inserted into the pPLEO1 vector (Alexeyev and Winkler, 1999), a derivative of pBluescript II SK(+) containing the *phoA-lacZ*α from pMA632. In order to generate the appropriate overhangs for exonuclease III nuclease digest, the pPLE01-*wzy*_β_ construct was digested with *Pst*I and *Xba*I. Once exonuclease III was added, aliquots were removed at 30 s intervals and put in a stop solution containing 100 mM EDTA, which resulted in a collection of *wzy*_β_ truncations of varying lengths. To generate blunt-ends, Mung Bean Nuclease was added to remove the 5′ overhangs and finally the Klenow fragment combined with dNTPs formed blunt-ends suitable for ligation and subsequent transformation into *E. coli* DH10B. To ensure proper coverage of the entire *wzy*_β_ sequence, site-targeted truncations were selected at 10 amino acid intervals and generated by amplifying fragments using site-specific primers to the desired terminal residue. These constructs were then digested with *Sac*I before inserting them into pPLE01. The random and site-targeted truncation library recoveries were plated onto dual-indicator plates containing 100 μg/ml ampicillin (Bio Basic), 1 mM isopropyl-β-D-thiogalactopyranoside (IPTG) (Bio Basic), 80 μg/ml 5-bromo-4-chloro-3-indolyl phosphate (BCIP) (Fisher), and 100 μg/ml 6-chloro-3-indolyl-β-D-galactoside (Red-Gal) (Research Organics). After incubation, the colonies were color scored based on the breakdown of the specific substrates: Red-Gal produced a red pigment indicative of β-galactosidase activity, BCIP produced a blue pigment indicative of alkaline phosphatase activity and a purple combination of both. To avoid full-length constructs and repeated truncation lengths, colony PCR was performed on all colored colonies with Taq polymerase (Life Technologies). Selected clones were sent for sequencing to identify the terminal residues of each truncation.

### Enzymatic assay

To assay the levels of enzyme activity, *E. coli* DH10B cells expressing the pPLEO1-*wzy*_β_ truncations were grown in triplicate overnight and subcultured in the presence of ampicillin. IPTG was added the morning after and the cells were grown to optical density at 600 nm (OD_600_) between 0.4 and 0.7. At this point, the culture was split into halves to assay for β-galactosidase using the Miller protocol and for alkaline-phosphatase following the Manoil protocol (Manoil et al., 1988). The normalized activity ratio (NAR) between these two enzymes was calculated as follows:

NAR = (alkaline phosphatase activity/highest alkaline phosphatase activity)/(β-galactosidase activity /highest β-galactosidase activity) (Lehane et al., 2005).

The resulting NAR determined the final localization and inputted into the HMMTOP 2.0 prediction algorithm (Islam et al., 2010).

### Site-directed mutagenesis

Site-directed mutagenesis (SDM) was performed using the QuikChange™ protocol (Agilent). Briefly, *wzy*_β_ was cloned into pHERD26T, a pBAD-based l-arabinose-inducible vector, and primers designed with the specific mutation were used to amplify using KOD Hot Start Polymerase (Novagen). Non-methylated parental DNA was digested using *Dpn*I (NEB) and the newly-formed nicked DNA was transformed into DH10B for sequencing (Table S1). In order to rapidly determine that mutant Wzy_β_ proteins do not demonstrate impaired membrane localization, the same mutations were generated within the pPLEO1-*wzy*_β_-T363 backbone, a periplasmic truncation, and grown on dual-indicator plates to determine whether they retained their periplasmic (blue) localization.

### Identifying a candidate *wzy_β_* promoter

The *wzy*_β_ sequence was localized within the recently published *P. aeruginosa* O2 genome (Thrane et al., 2015); however, the contig identified to contain the target ORF for *wzy*_β_ was misassembled as it was organized within three duplicated genes. Therefore, a new *de novo* genome assembly of O16 was performed using SPAdes (Bankevich et al., 2012). The resulting assembly graph was visualized using Bandage (Wick et al., 2015) and used for identification of the duplicated genes and their flanking scaffolds for manual assembly of the region. The identified scaffolds were then aligned to the sequence from a multidrug-resistant *P. aeruginosa* strain NCGM2.S1 (Miyoshi-Akiyama et al., 2011), which contains the D3 serotype converting unit, for ordering of the correct flanking regions and generating a consensus sequence. This newly formed superscaffold contained a sizeable sequence upstream of *wzy*_β._ Using this upstream nucleotide information as our reference sequence, a 1.65-kbp region containing the *wzy*_β_ and 500 bp upstream was amplified and then cloned into the mini-CTX2 integration plasmid for integration into *P. aeruginosa* cells (Hoang et al., 2000). The plasmid was transformed into *P. aeruginosa* using a standard electroporation protocol (Smith and Iglewski, 1989) and grown on LB-agar medium supplemented with 90 μg/ml tetracycline (Sigma). The unwanted backbone sequence was excised using the flippase encoded in the pFLP2 plasmid, which was then cured with growth on 5% sucrose (Hoang et al., 1998).

### Lipopolysaccharide complementation analysis

To characterize the *in vivo* function of both wild-type *wzy*_β_ and mutant *wzy*_β_, the plasmid containing each of these genes were transformed into a previously generated *wzy*_β_ chromosomal knockout (Kaluzny et al., 2007) and plated on LB agar medium supplemented with 90 μg/ml tetracycline. The additional serotypes analyzed in this study, including O1, O3, O5, O6, O8, O9, O10, O18, and O20 were grown overnight in the same LB medium. The transformation of pHERD26T-*wzy*_β_ was performed using the standard electroporation protocol and the transformants were plated onto LB agar supplemented with 90 μg/ml tetracycline. To observe varying OSA phenotypes, cultures were induced overnight with 0.1% l-arabinose, equilibrated the next morning to an OD_600_ of 0.45 and resuspended in Hitchcock and Brown lysis buffer for the preparation of LPS. The samples were heated at 100°C for 30 min and then treated with 5 μl of a 2 mg/mL proteinase K (Invitrogen) stock overnight at 55°C (Hitchcock and Brown, 1983). To observe OSA phenotypes, LPS was resolved by SDS-PAGE, and visualized based on silver staining and Western immunoblotting with monoclonal antibodies (mAb) specific to the serotypes. There are no monoclonal antibodies specific to serotype O18 and O20, therefore analysis of the OSA was done through silver staining and reactivity with the serotype O16 antibody.

## Results

### Wzy_β_ possesses an O-Antigen ligase superfamily motif

A characteristic of Wzy_α_ proteins is the high sequence variability resulting in a lack of conserved domains. However, a BLAST search using the Wzy_β_ amino acid sequence identified a conserved Wzy_C superfamily motif (residues 189–320) within the C-terminal half of the protein. The Wzy_C domain is a conserved motif found within the distal region of the O-Ag ligase superfamily and in PilO. Topology studies has localized this region of WaaL to the periplasm and mutagenesis data showed that residues within the domain are essential for function (Abeyrathne and Lam, 2007; Qutyan et al., 2007; Pérez et al., 2008). Further work by our group (Abeyrathne and Lam, 2007) showed that the His residue within this Wzy_C motif is essential for the function of O-antigen ligase WaaL. It is worth noting that all WaaL protein reported today perform inverting glycosyltransferase reactions (Schild et al., 2005; Abeyrathne and Lam, 2007; Ruan et al., 2012).

### Experimentally derived membrane topology Wzy_β_

**Figure 2:**
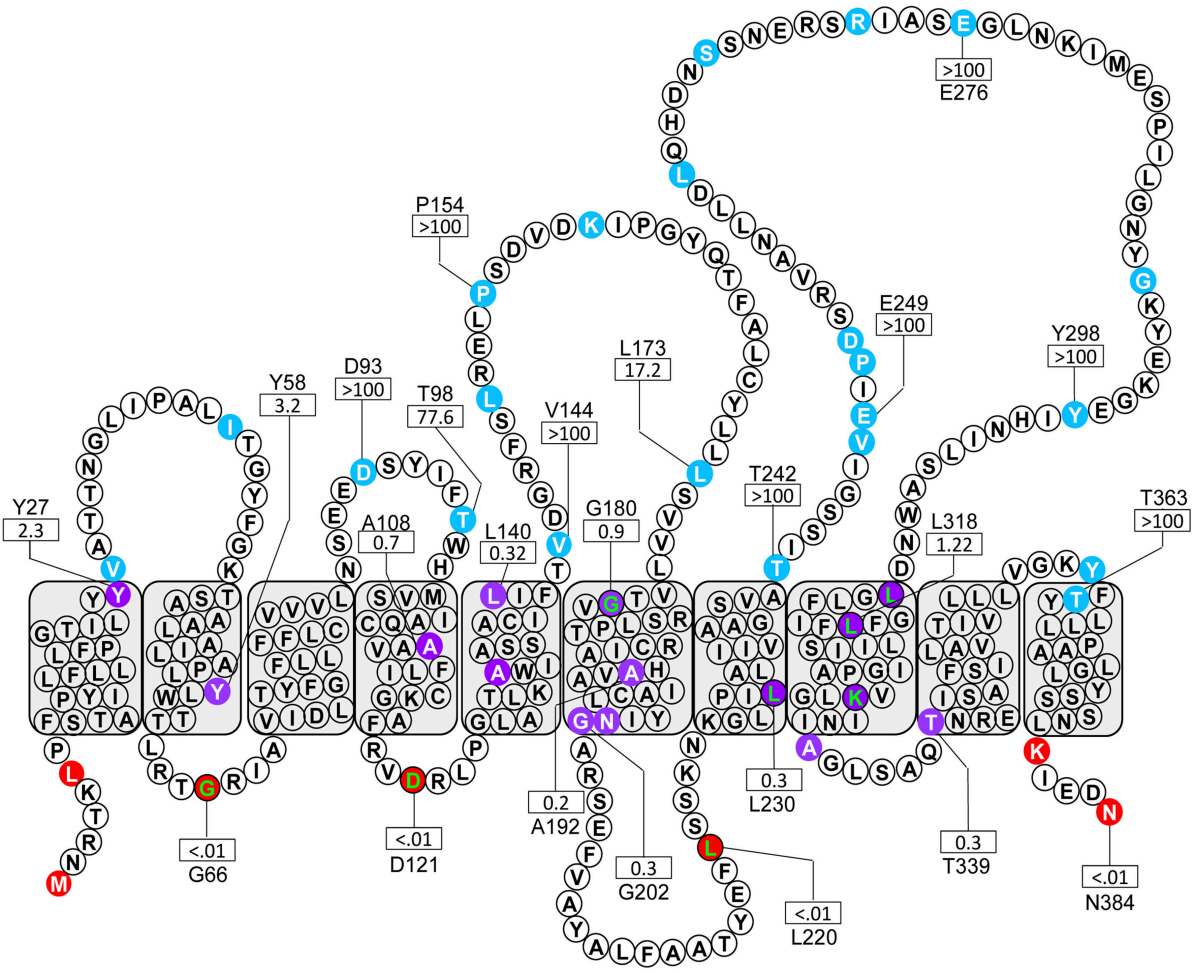
**Topological map of Wzy_β_ based on the localization of 24 amino acids obtained from a *phoA-lacZ*α fusion library**. The colored residues designate the subcellular localization of the given truncation: blue, periplasm, purple, TM; red, cytoplasm. The green lettered residues were generated through site-targeted fusions. The NAR values for the enzyme ratios are shown for selected residues by a rectangle.

A total of 51 truncations were generated using ExoIII digestion (Alexeyev and Winkler, 1999) resulting in 36 unique localizations (the colony morphology showed 22 blue, 10 purple and 4 red). To ensure a sufficiently complete coverage of Wzy_β_, seven site-targeted clones were generated resulting in three red and four purple colonies. Among the truncation constructs, 24 representative residues were selected for NAR evaluation: 17 from the random truncations and all seven of the site-targeted truncations (Table 1). Overall, the color scores of the truncations corresponded well with the anticipated enzyme values. The experimental-based topology map of Wzy_β_ hence developed is in stark contrast to the topology map derived from *in silico* analysis. The latter predicted that Wzy_β_ contained only a single large cytoplasmic loop between residues 246–302 (Figure S2). The experimental-based localizations were inputted into the HMMTOP 2.0 algorithm in order to produce the final map, which displayed the following structural features: (i) both the N- and C-terminal ends of Wzy_β_ are localized to the cytoplasm, (ii) 10 TMS span the length of the protein, (iii) two large periplasmic loops (PL) were observed, with PL3 spanning amino acids 142–178 and containing an RX_10_G motif and PL4 which spans amino acid positions 242–301 possesses an HX_9_G motif. The pI values are 4.68 for PL3 and 5.15 for PL4; and (iv) a cytoplasmic loop (CL) of considerable size was observed at amino acids 203–223 (Figure 2).

**Table 1:** **Normalized activities of AP and BG Wzy_β_ truncation fusions to PhoALacZα**.

**Residue^a^**	**Avg AP^b^**	**Avg BG^c^**	**%AP^d^**	**%BG^e^**	**NAR (%AP/%BG)^f^**	**Colony Color^g^**	**Localization^h^**
**RANDOM**							
Y27	16.2	49	100	66.5	2.3	Blue	TM
Y58	2.3	4.1	13.8	5.5	3.2	Blue	TM
D93	8.7	−0.38	53.3	−0.5	>100	Blue	P
T98	12.9	0.7	79.9	1.03	77.6	Purple	P
A108	3.6	22	22.4	29.9	0.7	Purple	TM
L140	3.7	53	23.1	71.9	0.32	Purple	TM
V144	1.7	−4.1	10.3	−5.6	>100	Blue	P
P154	8.1	−8.7	49.6	−11.7	>100	Blue	P
L173	3.2	0.9	19.8	1.15	17.2	Purple	P
A192	3.2	73.7	19.8	100	0.2	Purple	TM
G202	5.3	69.5	32.3	94.1	0.3	Blue	TM
T242	8.2	−2.3	50.4	−3.1	>100	Blue	P
E249	10	0.3	61.5	0.4	>100	Blue	P
E276	4.8	−5.9	29.7	−8	>100	Blue	P
Y298	7.5	−1.3	46.5	−1.76	>100	Blue	P
T339	1.0	15.16	6.3	20.5	0.3	Purple	TM
T364	2.48	−0.9	15.3	−1.1	>100	Blue	P
**SITE TARGET**							
G66	−2.7	27.7	−30.1	25.9	< 0.01	Red	C
D121	−0.7	76.5	−6.4	67.5	< 0.01	Red	C
G180	5.7	40.1	63.7	35.5	0.95	Purple	TM
L220	−6.4	66	−61.1	61.7	< 0.01	Red	C
L230	0.8	32.6	8.96	30.5	0.29	Red	TM
L318	0.6	6.4	7.25	5.9	1.22	Purple	TM
N384	−0.8	107	−8.67	100	< 0.01	Red	C

*Localization of wzy_β_ truncations based on the enzymatic ratio between alkaline phosphatase (AP) and β-galactosidase (BG)*.

a *Location of the terminal residue followed by the reporter*.

b, c *AP and BG activity quantified in Miller units as described in the Materials and Methods*.

d, e *Percentage of AP and BG activities of each fusion in relation to the maximum measured activity*.

f *Normalized %AP / % BG activity ratio (NAR)*.

g *Color score of expressed clones grown on dual-indicator plates*.

h *Final localization of terminal residues used to generate the topology map: periplasm (P), cytoplasm (C), transmembrane (TM)*.

### Positively-charged residues localized within PL3 are essential for Wzy_β_ function

**Figure 3:**
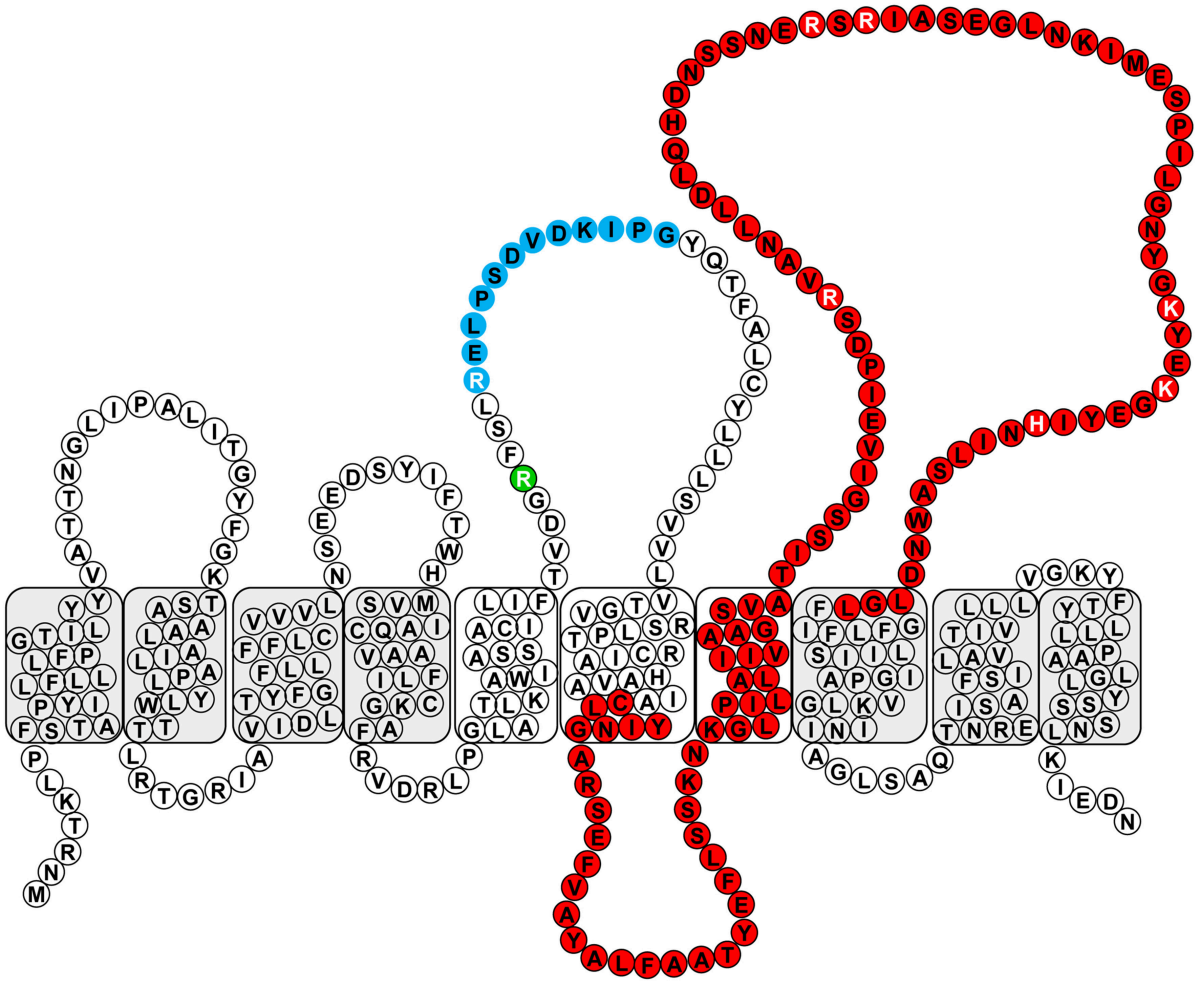
**Conserved domains within Wzy_β_**. The RX_10_G motif is depicted in blue, and the Wzy_C domain is depicted in red. The residues selected for mutagenesis are written in white font and R147 is depicted in green.

**Figure 4:**
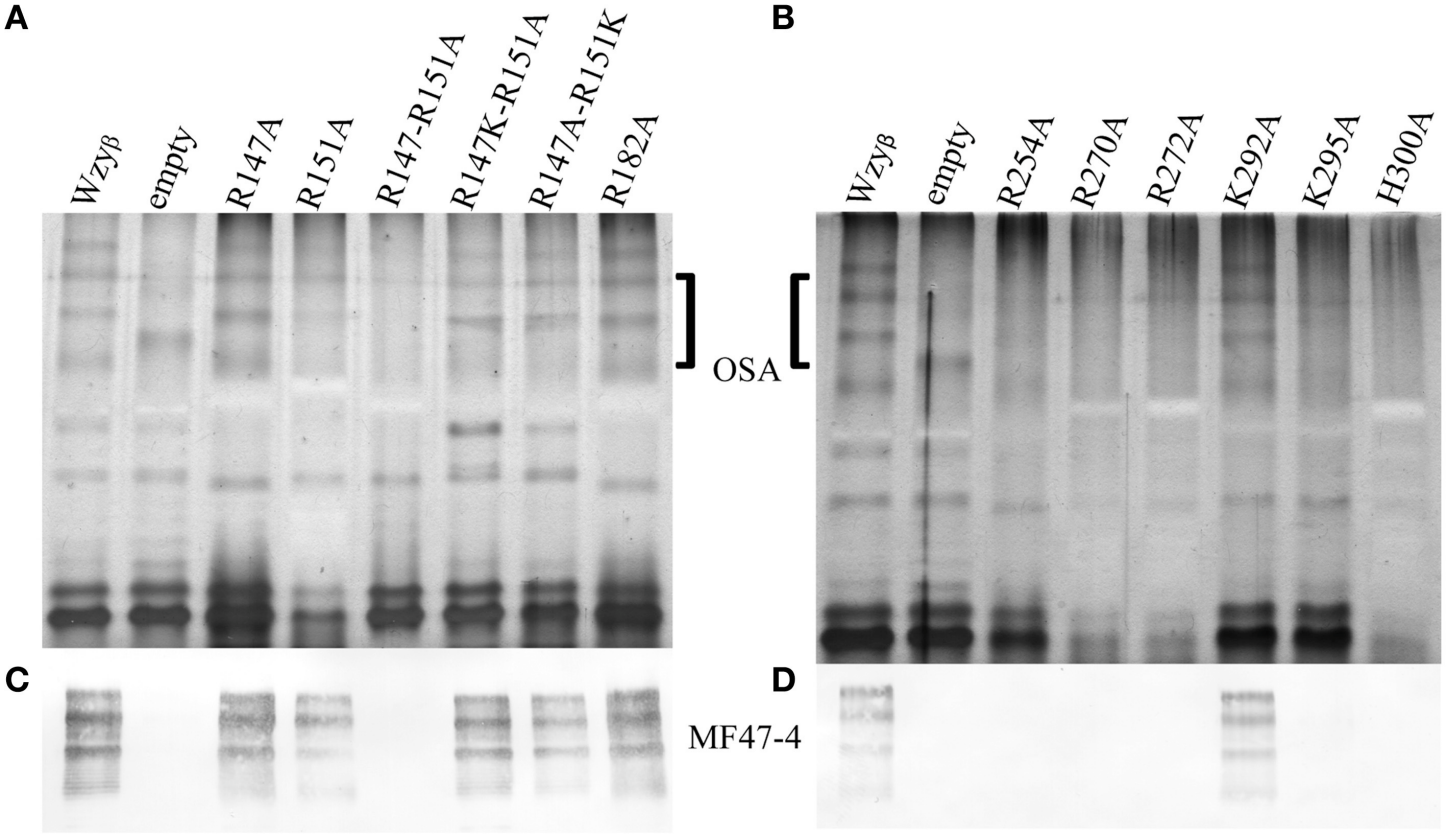
**SDS-PAGE and Western immunoblotting analyses of LPS isolated from *wzy*_β_::Gm^r^ complemented with Wzy_β_ mutants generated through alanine-scanning mutagenesis. (A)** Silver stain of residues localized in PL3. **(B)** Silver stain of residues localized in PL4. The OSA region is highlighted. **(C,D)** Western immunoblots probed with monoclonal antibodies to serotype O16 OSA (MF47-4) (Lam et al., 1987).

The experimentally-derived topology map revealed the presence of Arg residues within PL3 at positions 147 and 151, which make up the conserved RX_10_G motif, and an additional Arg at position 182 (Figure 3). Neither of the SDM mutants, R147A nor R151A, showed a disruption in OSA biosynthesis, though the latter mutation, R151A, caused a reduction in the level of OSA being produced. However, a double-mutation construct, R147A-R151A, exhibited a distinct lack of β-linked OSA (Figures 4A,C). Since, the guanidinium side group of Arg residues has been shown to be associated with carbohydrate binding (Dahms et al., 1993), we set out to screen whether positive-charge alone would suffice for function of the Wzy_β_ protein at these locations by producing R147K and R151K substitutions. In both cases, wild-type level of OSA was restored, demonstrating that a positive charge in PL3 is important for Wzy_β_ activity. (Figures 4A,C). Although the R147A-R151A is a double mutant, generating these mutations in the T363 truncation background did not affect periplasmic localization as evidenced by the blue coloration of the bacterial colonies on the dual-indicator plates. This observation indicated that the mutant version of Wzy_β_ was inserted into the IM (Figure S3).

### PL4 of Wzy_β_ contains residues essential for an inverting glycosyltransferase reaction

**Figure 5:**
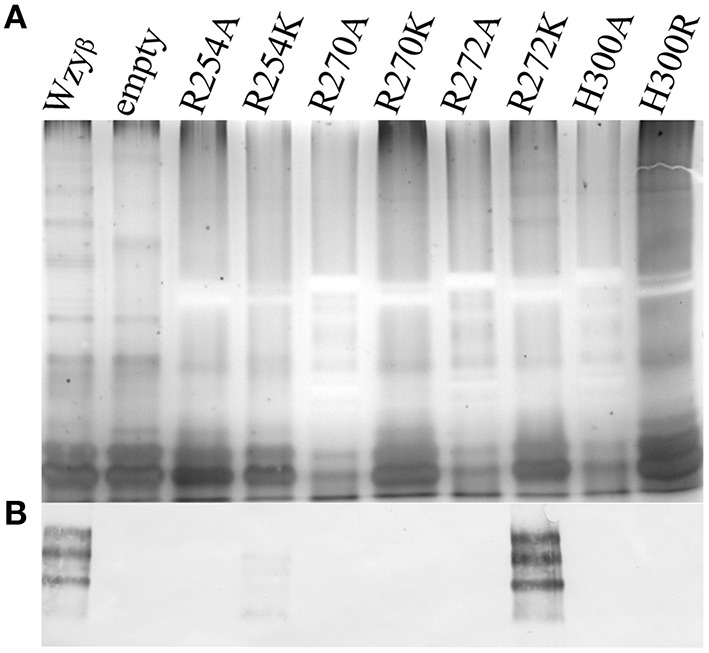
**Analysis of LPS from *wzy*_β_::Gm^r^ complemented with like-charge residues by SDS-PAGE and Western immunoblot. (A)** Silver stain with the OSA region highlighted and **(B)** Western immunoblot probed with monoclonal antibodies to serotype O16 OSA (MF47-4) (Lam et al., 1987).

To establish that the Wzy_C domain (Figure 3) plays a significant role in Wzy_β_ activities and to identify amino acid residues that might be important for function, we decided to do alanine-scanning mutagenesis and substituted conserved residues throughout PL4 with Ala. These included Arg^254^, Arg^270^, Arg^272^, Lys^292^, Lys^295^, and His^300^. Note that H300 is an essential residue in the HX_10_G motif. When the SDM mutant constructs were expressed in the *wzy*_β_ knockout, all but K292A demonstrated impaired Wzy_β_ function (Figures 4B,D). Further investigation using like-charge substitutions revealed that the guanidinium side-group at positions Arg^254^ and Arg^270^ are important for Wzy_β_ activity, as evidenced by the inability of R254K and R270K to complement function of the *wzy*_β_ knockout mutant (Figure 5). Unlike the findings in the mutagenesis of WaaL of *P. aeruginosa*, a H300R substitution was unable to complement the *wzy*_β_ knockout and restore polymerase function. The mutants generated within the T363 truncation construct retained their periplasmic localization; hence the charged amino acids selected for mutagenesis within this region (250–363) of the protein are apparently not required for protein folding/membrane insertion (Figure S3).

### Identification of the Wzy_C domain in heterologous Wzy proteins

We were able to take advantage of the increase in available information on O-Ag polysaccharide structures and O-Ag gene cluster sequences in public databases to investigate the prevalence of the Wzy_C domain within known Wzy amino acid sequences that we extracted from GenBank. This bioinformatic exercise revealed that a relationship between O-Ag structures and the occurrence of Wzy_C domain in Wzy homologs was conserved in 82% of the cases when the corresponding O-Ag structure is β-linked. Importantly, it was noted that the Wzy_C domain is absent in the amino acid sequences of Wzy proteins associated with α-linked O-Ag structures (Table 2).

**Table 2:** **Occurrence of the Wzy_C domain in organisms with β-linked O-Ag**.

**Organism**	Intramolecular linkage^*^	Wzy_C^**^	**References**	**GenBank**
*P. aeruginosa* O5	(β1 → 3)-D-FucNAc-(**α1 →**	No	de Kievit et al., 1995	AAM27801.1
*P. aeruginosa* O16	(β1 → 3)-D-FucNAc-(**β1 →**	Yes	Kaluzny et al., 2007	ABM21470.1
*P. aeruginosa* O9	(α1 → 3)-D-QuiNAc(**β1 →**	No	Raymond et al., 2002	AAM27879.1
*P. aeruginosa* O13	(α1 → 3)-D-QuiNAc-(**β1 →**	Yes	Raymond et al., 2002	AAM27615.1
*P. aeruginosa* O3	(α1 → 3)-D-QuiNAc4N*S*Hb-(**β1 →**	Yes	Raymond et al., 2002	AAM27766.1
*P. aeruginosa* O1	(α1 → 3)-D-QuiNAc(**α1 →**	No	Raymond et al., 2002	AAM27546.1
*Escherichia coli* O86:B7	(β1 → 3)-D-GalNAc(**α1 →**	No	Yi et al., 2006	AY220982.1
*Shigella flexneri*	(β1 → 3)-D-GlcNAc(**α1 →**	No	Morona et al., 1994; Knirel et al., 2013	CAA50774.1
*Francisella tularensisi*	(β1 → 2)-Qui4NFm(**α1 →**	No	Vinogradov et al., 1991; Kim et al., 2010	ABU61108.1
*Streptococcus pneumoniae* 9a	(β1 → 4)-Glc(**β1 →**	Yes	Bentley et al., 2006; Aanensen et al., 2007	CAI32973.1
*Salmonella anatum*	(1 → 4)-Rha-(**α1 →**	No	L'Vov et al., 1989	AHW12776.1
*Epsilon 15*	(1 → 4)-Rha-(**β1 →**	Yes	Robbins et al., 1965; Kropinski et al., 2007	AAO06084.1
*Yersinia pseudotuberculosis* O:2a	(β1 → 3)-D-GlcNAc(**α1 →**	No	Kondakova et al., 2008; Kenyon and Reeves, 2013	AAN23078.1
*Klebsiella pneumoniae* K57	(1 → 2)-α-Man-(**β1 →**	Yes	Kamerling et al., 1975; Pan et al., 2008	BAF75760.1
*Vibrio vulnificus* 27562	(1 → 4)-β-L-Rha(**β1 →**	Yes	Gunawardena et al., 1998; Nakhamchik et al., 2007	ADO64242.1
*Streptococcus pneumoniae* 4a	(α1 → 3)GalNAc(**α1 →**	No	Bentley et al., 2006	CAI32772.1
*Shigella boydii*	(β1 → 3)-D-GlcNAc-(**α1 →**	No	Tao et al., 2004	AAS98031.1
*Acetinobacter baumannii*	(β1 → 3)-D-GalNAc(**β1 →**	No	Kenyon and Reeves, 2013	AHM95427.1
*Staphylococcus aureus* O5	(β1 → 3)-d-Fuc*p*NAc-(**β1 →**	Yes	Moreau et al., 1990	AAC46093.1
*Burkholderia cepacia* K56-2	(α1 → 3)-GalNAc(**β1 →**	Yes	Varga et al., 2013	EPZ86155.1

* *The orientation of the intramolecular bond is indicated in bold*.

** *Presence and absence of Wzy_C domain in the Wzy homolgos of various species and bacterial strains*.

### Wzy_β_ is specific to the O2 serogroup OSA structures

**Figure 6:**
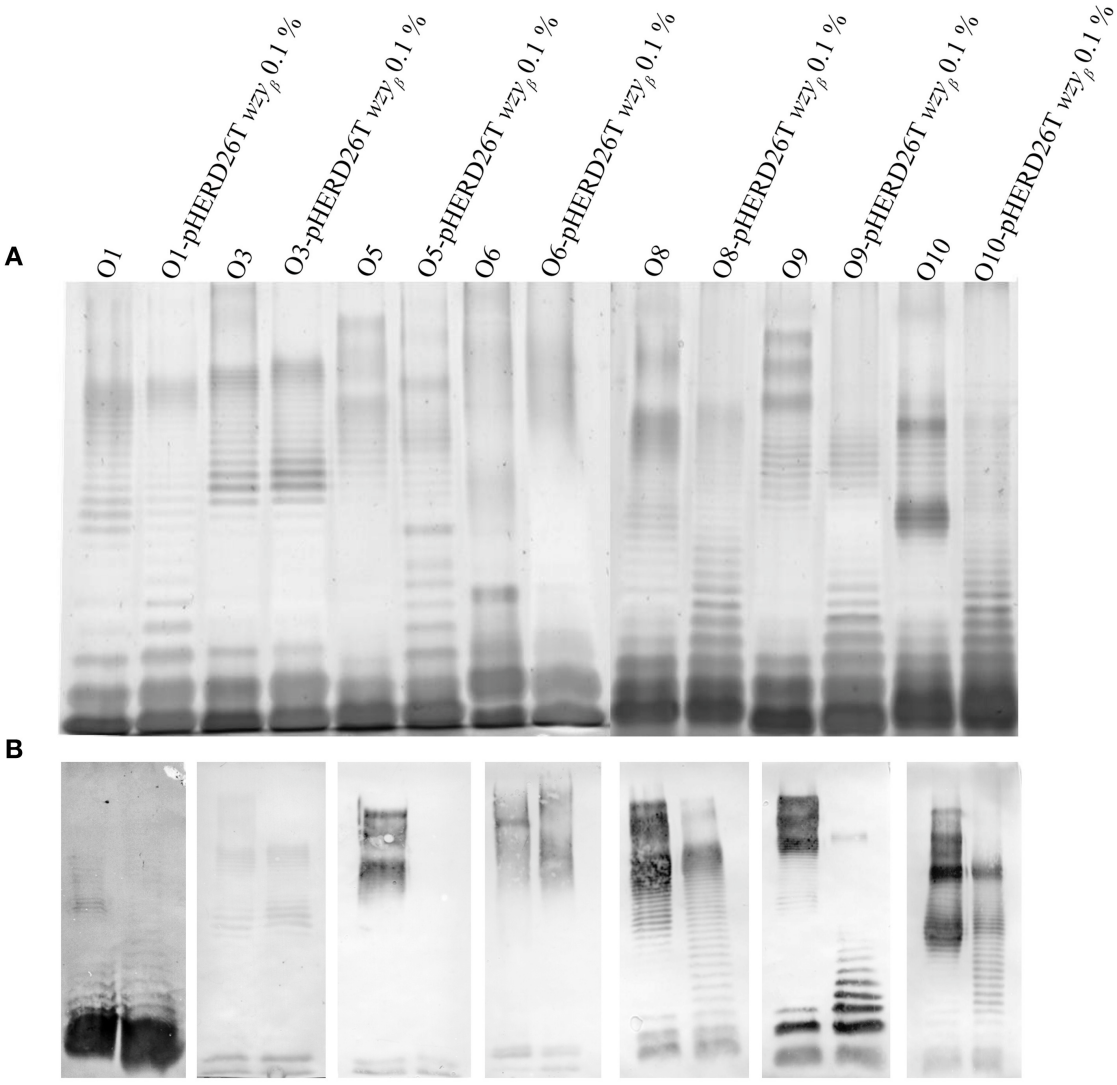
**Wzy_β_ expression in various *P. aeruginosa* serotypes**. LPS harvested from wild-type O1, O3, O5, O8, O9 and O10 and expressing the pHERD26T-*wzy*_β_ with 0.1% l-arabinose construct were electrophoresed by SDS PAGE and analyzed by **(A)** silver staining and **(B)** Western immunoblot of serotypes with antibodies specific to O1 (MF25-1), O3 (MF5-4), O5 (MF15-4), O8 (MF30-1), O9 (MF43-1), and O10 (MF54-1), and inner core 5c-7-4 (Lam et al., 1987).

**Figure 7:**
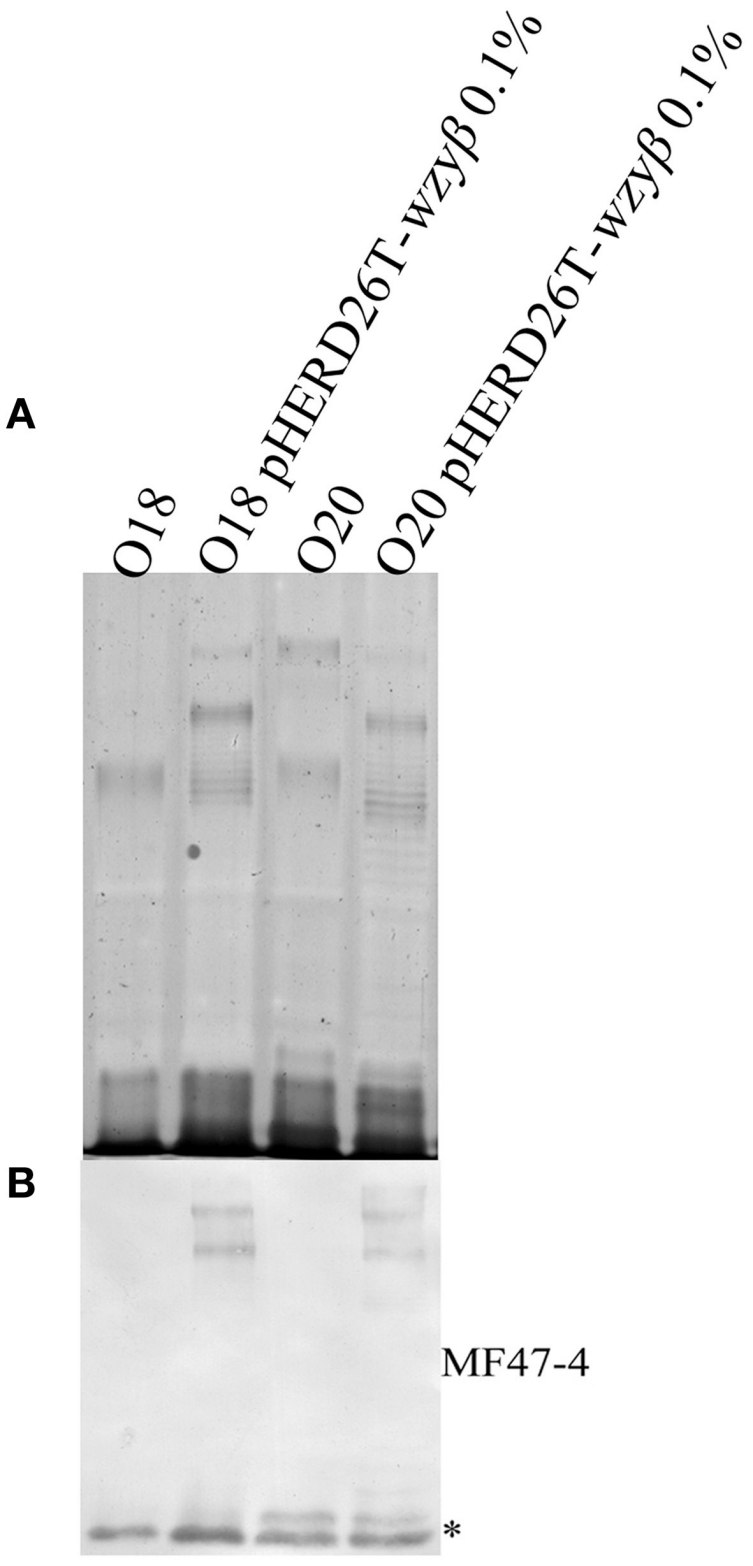
**Expression of Wzy_β_ in serotype O18 and O20**. **(A)** Silver staining and **(B)** Western immunoblot (MF47-4, O16-specific) of LPS harvested from wild-type O18 and O20 and expressing pHERD26T Wzy_β_ with 0.1% l-arabinose.

As Wzy_β_ was acquired from a yet unknown environmental source, it may have relaxed specificity to other O-antigen repeats. Wzy_β_ was therefore expressed in non-O5 serotypes, i.e., O1, O3, O6, O8, O9, and O10. The most obvious changes were observed in the serotypes which produce trisaccharide repeats: O8, O9, and O10 wherein the presence of Wzy_β_ affects the OSA biosynthesis as evidence by altered modal lengths in O8 and O10 and complete disruption of the native OSA in serotype O9 (Figure 6). The altered interaction between the native Wzy and Wzz demonstrates that Wzy_β_ localizes to their OSA biosynthesis machinery. This phenomenon is not observed when Wzy_α_ from serotype O5 is expressed in serotype O9, which is consistent with Wzy_α_ being highly specific to its native OSA structure (Figure S4). However, no evidence of altered intramolecular linkages was observed, demonstrating that although it can recognize the machinery, Wzy_β_ cannot utilize non-O2 serogroup OSA repeats. As a control for O2 serogroup specificity, the pHERD26T-*wzy*_β_ construct was expressed in O18 and O20 as both are members of the O2 serogroup. This heterologous expression resulted in the bacteria producing β-linked OSA (Figure 7).

### *P. aeruginosa* PAO1 can simultaneously produce OSA chains with distinct intramolecular linkages

**Figure 8:**
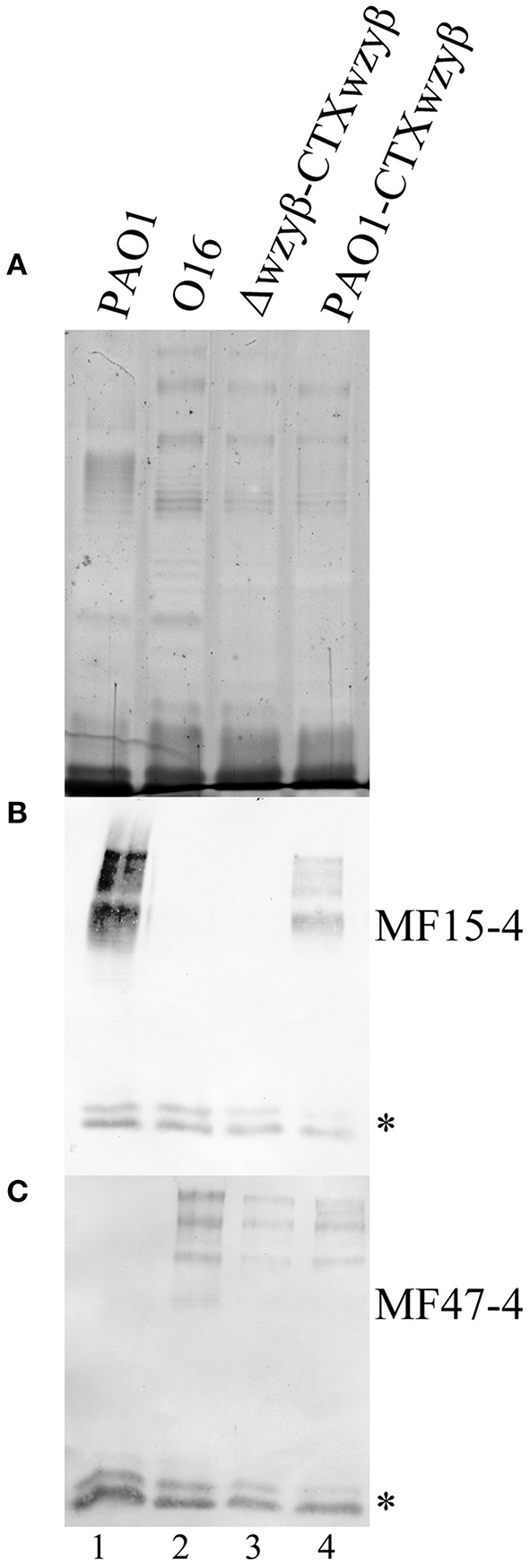
**Analysis of the OSA structures after Wzy_β_ integration into the PAO1 chromosome**. **(A)** Silver stain and Western immunoblot probed with mAbs [MF15-4, O5-specific **(B)**, and MF47-4, O16-specific **(C)**]. mAb 5c-7-4 (specific to the inner core) was used as an internal standard for loading, and low molecular weight bands reacting to 5c-7-4 are inner core LPS bands and these are designated with ^*^. The lanes are numbered 1 to 4.

*Wzy*_β_ and its native promoter were successfully amplified from the O16 chromosome based on the reconstruction of an O2 isolate genome utilizing the SPAdes system, confirming that these serotypes share an identical serotype-converting unit. The integration of *wzy*_β_ driven by its native promoter into the PAO1 chromosome using the miniCTX system resulted in the presence of two distinct OSA banding patterns as detected by silver staining (Figure 8A) and Western immunoblotting with serotype-specific mAbs, MF15-4 (O5 specific) (Figure 8B), and MF47-4 (O16-specific) (Figure 8C). The control lanes 1 and 2 demonstrate that PAO1 and serotype O16 produce α-linked and β-linked OSA repeats respectively. Lane 3 is the *wzy*_β_ knockout complemented with miniCTX2-*wzy*_β_ and the candidate promoter as indicated by the presence of β-linked OSA repeats. Therefore the included upstream region, identified during chromosomal reconstruction, must contain a promoter for *wzy*_β_ as the miniCTX system does not have a plasmid encoded promoter region. Lane 4 depicts LPS harvested from PAO1 that has the miniCTX2-*wzy*_β_ integrated within its chromosome. The presence of signal in both the O5-specific (B) and O16-specific (C) Western immunoblots is indicative of concomitant expression of the cognate Wzy_α_ and the phage-derived Wzy_β_. Hence two separate OSA polysaccharides α-linked and β-linked were produced in the same cell population (Figure 8).

## Discussion

The mechanism used by the integral IM protein Wzy to polymerize long-chain OSA is currently unknown. The highly diverse *P. aeruginosa* OSA structures that make up the various serotypes provides an opportunity to study conserved features and differences among the membrane proteins associated with the assembly of LPS. A major hurdle to the investigation of the O-polymerase-mechanism is the lack of any published high-resolution structural data of Wzy proteins. Previous work in our lab established the genetic basis for the role of the phage-derived polymerase, Wzy_β_, a component of the serotype-conversion locus in the D3 genome responsible for lysogenic conversion from serotype O5 to O16. (Newton et al., 2001; Kaluzny et al., 2007); however, no further investigations have been undertaken so far to decipher its function. In this study, we used the *phoA-lacZ*α dual-reporter system and obtained experimental data to help elucidate the membrane topology of Wzy_β_.

Unlike previous experimental investigations on Wzy (Daniels et al., 1998; Islam et al., 2010; Kim et al., 2010), a BLAST search has led to a novel observation that the Wzy_β_ sequence contains a Wzy_C domain, also called an O-Ag ligase domain due to its prevalence in WaaL proteins found in various Gram-negative species (Marchler-Bauer et al., 2013). In the case of Wzy_β_, the Wzy_C domain spans amino acid residues 234–310. Importantly, this stretch of amino acids is conserved among members of the WaaL family of proteins, which are known to catalyze an inverting glycosyltransferase reaction to link O-Ag to the lipid A-core in a proposed metal-independent reaction (Schild et al., 2005; Abeyrathne and Lam, 2007; Ruan et al., 2012). It was observed that OSA trisaccharide units of *P. aeruginosa* PAO1 are linked via an α-bond to Und-P, their lipid-carrier, therefore Wzy_β_ must perform an inverting glycosyltransferase reaction to OSA repeats that confer β-linked intramolecular bonds in serotype O16 (Rocchetta et al., 1998; Sadovskaya et al., 2000; Bystrova et al., 2006; Woodward et al., 2010). To determine whether this domain is essential for Wzy_β_, a topology map needs to be established such that appropriate experiments can be designed to probe the roles of specific amino acid residues and any potential functional motifs that might be involved in functional activities of this β-polymerase.

The existing topology maps of WaaL proteins all showed that the Wzy_C domain contains an essential His residue near the distal portion of the protein that forms a characteristic large periplasmic loop (Schild et al., 2005; Abeyrathne and Lam, 2007; Pérez et al., 2008; Islam et al., 2010). As such, the *de novo* topology map that was easily generated by using the *in silico* algorithms TOPCONS and HMMTOP 2.0 of Wzy_β_ was deemed unsatisfactory to utilize for downstream studies because two large periplasmic loops were absent; hence, this deficiency provides the rationale for developing an experimentally-based topology of Wzy_β_ as the polymerase reaction occurs in the periplasm (Figure S2) (Robbins et al., 1967; Whitfield, 1995). Our group has successfully used the *phoA-lacZ*α dual-reporter system to determine the topology of Wzx, Wzy_α_, and WaaL from *P. aeruginosa* strain PAO1 (Islam et al., 2010). The knowledge of the topology of these membrane proteins is pivotal to our ability to initiate protein modeling (Islam et al., 2012) and biophysical experiments (Islam et al., 2013b) in order to examine the function of Wzx of *P. aeruginosa*. As a case in point, our group was able to develop a tertiary model of Wzx (Islam et al., 2012), generated based on comparison to the crystal structure of the closely-related protein called NorM of *Vibrio cholerae* (He et al., 2010). The topology of NorM showed strong similarity to the dual-reporter generated topology of Wzx, therefore gaining our confidence in the ability of this strategy to accurately localize particular domains in these membrane proteins (Islam et al., 2012). Although the presence of a prominent cytoplasmic loop was novel, no mutagenesis experiments have been pursued due to the previous report demonstrating that the Wzy protein of *E. coli* does not hydrolyze ATP (Woodward et al., 2010).

Based on the importance of the PL domains in Wzy_α_ participating in the proposed “catch-and-release” mechanism of O-Ag polymerization, we undertook this project to obtain structure-function data of Wzy_β,_ a completely unrelated polymerase; our findings in this study provide further evidence for a conserved polymerase topology regardless of whether the resultant bond between O-units is α or β due to the activities of these two classes of Wzy proteins. Upon investigation of the net-charges across both Wzy_β_ PL regions, no particular amino acid stretch with notable positive charge was found within PL3 of Wzy_β_, rather, a net-negative charge was seen. This does not discredit the catch-and-release mechanism as both the *Shigella flexerni* and *Francisella tularensis* Wzy proteins have a negatively-charged PL3 but in the case of *S. flexneri* the Arg residue in the noted RX_15_G motif was found to be essential for function (Kim et al., 2010; Nath and Morona, 2015). Previously, our lab used a position-based algorithm, *Jackhmmer* (Finn et al., 2011) and discovered distant homologs of Wzy_α._ Using this bioinformatic software alleviates the hurdle caused by the absence of strong sequence similarity among these proteins (Islam et al., 2013a). In the current study, the sequence alignment analysis using *Jackhmmer* was re-run, but this time, we included the peptide sequence of PL3 of Wzy_β_. As expected, the RX_10_G motif in PL3 (R151) of Wzy_β_ aligned with the PL3 sequences from a number of other Wzy proteins that were not identified in the previous report (data not shown). Therefore, PL3 possesses essential positively-charged amino acids throughout which includes a conserved Arg residue that was identified in a large sequence alignment of heterologous Wzy-like proteins. With the observation of essential positive charge in PL3 of Wzy_β_, it lends support to the role of a proximal periplasmic loop being utilized for substrate recruitment (Islam et al., 2011, 2013a; Nath and Morona, 2015).

A hallmark of the O-Ag ligase motif (Wzy_C) is the presence of a basic residue that is essential for inverting glycosyltransferase activities which serves to stabilize the leaving phosphate group once the transferase reaction occurs (Chiu et al., 2004; Lairson et al., 2008; Ruan et al., 2012). In the case of *P. aeruginosa* WaaL and Wzy_β_, the residue in question is His of the HX_10∕9_G motif; a null mutation of this residue abrogates the function of these proteins (Abeyrathne and Lam, 2007; Lairson et al., 2008; Ruan et al., 2012). Although Wzy_β_ contains this motif, it is unable to be used to complement a *waaL*::Gm^r^ mutant in a complementation assay (data not show). There could be a number of reasons why this complementation did not work, one of them being that WaaL and Wzy are vastly different proteins; although both share the HX_10∕9_G motif, cross complementation of the ligase reaction with a polymerase protein is not a natural phenomenon. However, the negative complementation outcome, could also suggest that the motif in Wzy_β_ is not involved in substrate recognition as in WaaL proteins, as the latter have high specificity in the recognition of lipid A-core as part of the substrate. A line of evidence that supports the interpretation that the presence of this motif attributes to inverting glycosyltransferase activities in Wzy polymerases was obtained from BLASTp analyses of a number of heterologous Wzy sequences from bacterial species with published O-Ag structures. With this exercise, the Wzy_C domain was identified in 9 of the 11 Wzy proteins from organisms that have corresponding β-linked O-Ag structures (82%).

Two Wzy sequences from organisms, which produce β-linked O-Ag but do not contain the Wzy_C domain include: Wzy from *P. aeruginosa* serotype O9 (Wzy_PaO9_) and *Acinetobacter baumannii* (Wzy_Acb_). These two proteins show higher levels of similarity to the “EspG” superfamily of membrane-bound glycosyltransferases than to other Wzy sequences. This is puzzling because EpsG family proteins have been reported to function as autophosphorylating tyrosine kinases, similar to Wzc, which are involved in chain-length regulation of bacterial capsules (Paiment et al., 2002; Ayabe-Chujo et al., 2012). Future work to investigate into the recently published O9 genome may reveal a previously uncharacterized Wzy (Thrane et al., 2015).

Of particular note is the presence of the Wzy_C domain in Wzy_β_ from the seroconverting unit of ϵ15, a bacteriophage that targets *Salmonella* species, which when infected with a phage would undergo lysogenic conversion through expression of an inhibitor of α-polymerase (Uetake et al., 1958; Losick, 1969). Hence, ϵ15 likely causes serotyping switch in *Salmonella* species via a similar mechanism as that observed in D3 bacteriophage, which causes serotyping switch of *P. aeruginosa* serotype O5 to O16 (Newton et al., 2001). Therefore it can be reasoned that the polymerase gene within the serotype converting units would perform a different mechanism (inverting) than the cognate Wzy_α_ (retaining) to ensure resistance to the α-polymerase inhibitor. Unimpeded Wzy_β_ activity would lead to long-chain OSA formation with β-bonds between O-units and provide resistance to subsequent phage infection (Losick, 1969; Barksdale and Arden, 1974; Taylor et al., 2013). Through SDM experiments, our data showed that the distal arm of Wzy_β_ (PL4) contains essential residues, Arg^270^ and His^300^, which is conserved in the Wzy_C domain as the proposed stabilizing base. It is conceivable that these residues are involved in the transferase reaction. The consistent presence of this His residue in other β-polymerases suggests that this particular region of the protein could be the determinant of bond formation. The inability of H300R to restore glycosyltransferase function may be due to the essential nature of the imidazole ring as seen in GT-1 metal-independent glycosyltransferases where His acts as the base (Brazier-Hicks et al., 2007; Lairson et al., 2008).

The observation that Wzy_β_ is able to polymerize OSA repeats despite the presence of the cognate Wzy_α_ helps to better understand the mechanism of serotype conversion. Unlike Wzy_α_ from PAO1, the ability of Wzy_β_ to localize and interfere with the native Wzy/Wzz interaction in non O2 serogroup serotypes, demonstrates its relaxed specificity toward the OSA biosynthesis/assembly machinery. With the ability of Wzy_β_ to disrupt the cognate Wzy-Wzz interaction, we propose that both Wzy_β_ and Iap work in concert and not sequentially. If these two proteins worked sequentially, Wzy_β_ would not be able to polymerize OSA repeats while Wzy_α_ was still functional. Once Wzy_β_ was expressed at native levels, it is apparent that the total amount of OSA is split between α- and β-linked polymers as indicated by the relative intensity of the OSA-LPS bands in lane 4 (Figure 8) compared to the WT O5 and O16. Our interpretation is that the Iap, which also affects the Wzz/Wzy_α_ interaction (Taylor et al., 2013), must non-specifically localize to the OSA biosynthetic machinery, where it inhibits Wzy_α_ but is unable to affect Wzy_β_, which we propose is due to its differing glycosyltransferase mechanism. This is supported by the observation that the total amount of β-linked OSA produced never reaches the levels of WT α-linked OSA in serotype O5 even when Wzy_β_ is overexpressed on a plasmid. We propose that OSA repeats are still interacting with Wzy_α_ and are therefore unable to enter in contact with the functional Wzy_β_ to be polymerized. The phenomenon of bacteria producing multiple OSA phenotypes on the cell surface is not uncommon within the context of O-Ag diversity as *P. aeruginosa* and, recently, *Azosparillum* and *Salmonella* have demonstrated concomintant expression of multiple O-Ag phenotypes (Knirel et al., 1982; Nghiêm et al., 1992; Sigida et al., 2015). Further, the ability of Wzy_β_ to alter the chain-length modality in serotype O5, O18, and O20 demonstrates that intra-molecular linkages affect OSA modal lengths.

In conclusion, the use of a dual-reporter approach to develop a topology map of Wzy_β_ revealed that it possesses two predominant PL loops, a structural feature we now confirm to be shared by other Wzy proteins. Another significant finding is the identification of essential amino acid residues within PL3 and in PL4. Through SDM and complementation experiments, we identified the amino acids, Arg^270^ and His^300^ that are associated with inverting glycosyltransferase activities of Wzy_β_. Finally, we have obtained evidence to show that the two periplasmic loops are important structural characteristics of Wzy proteins, from both α- and β- families of these proteins. These new evidence is consistent with the proposed “catch-and-release” mechanism of substrate shuttling. The two periplasmic loops are purported to participate in substrate shuttling, despite catalyzing the formation of different stereochemical linkages. Future investigations into polymerase function could include obtaining three-dimensional structures of PL4 from Wzy_β_ and PL5 from Wzy_α_ for an in-depth structure-function comparative study. This mechanistic difference between polymerase proteins further sheds light on the phenomenon of serotype conversion, a cause of O-Ag diversity in Gram-negative organisms.

## Author contributions

VT designed the research and wrote the paper; VT, JH, ST and SH all contributed to executing experiments, VT, ST, and LJ performed data and bioinformatics analyses. JL and all other authors also contributed to writing, editing, and approval of the final manuscript.

### Conflict of interest statement

The authors declare that the research was conducted in the absence of any commercial or financial relationships that could be construed as a potential conflict of interest.
