# Supplementary material for: A Bacteriophage-Acquired O-Antigen Polymerase (Wzyβ) from P. aeruginosa Serotype O16 Performs a Varied Mechanism Compared to Its Cognate Wzyα
Source: Front Microbiol. 2016 Mar 31;7:393. doi: 10.3389/fmicb.2016.00393 (PMC4815439; doi:10.3389/fmicb.2016.00393)
Supplement: Supplementary file 1 [file DataSheet1.docx]

**A bacteriophage-acquired O-antigen polymerase (Wzy_β_) from *P. aeruginosa* serotype O16 possesses conserved domains but a varied glycosyltransferase mechanism as compared to the cognate Wzy_α_ of serotype O5.**

Véronique L. Taylor^1^, Jesse P. Hoage^1^, Sandra Wingaard Thrane^2^, Steven M. Huzczsynski^1^, Lars Jelsbak^2^ and Joseph S. Lam^1^*
^1^ Department of Molecular and Cellular Biology , University of Guelph, Guelph, Canada
^2^ Department of Systems Biology, Technical University of Denmark, Kongens Lyngby, Denmark

(short title: bacteriophage polymerase mechanism of function resists inhibition)

*Corresponding author:

Joseph S. Lam: Email address: [jlam@uoguelph.ca](mailto:jlam@uoguelph.ca): Tel: 519-824-4120 x58332

**Figure S1 Alignment of Wzy_α_ and Wzy_β_ from *P. aeruginosa***

**
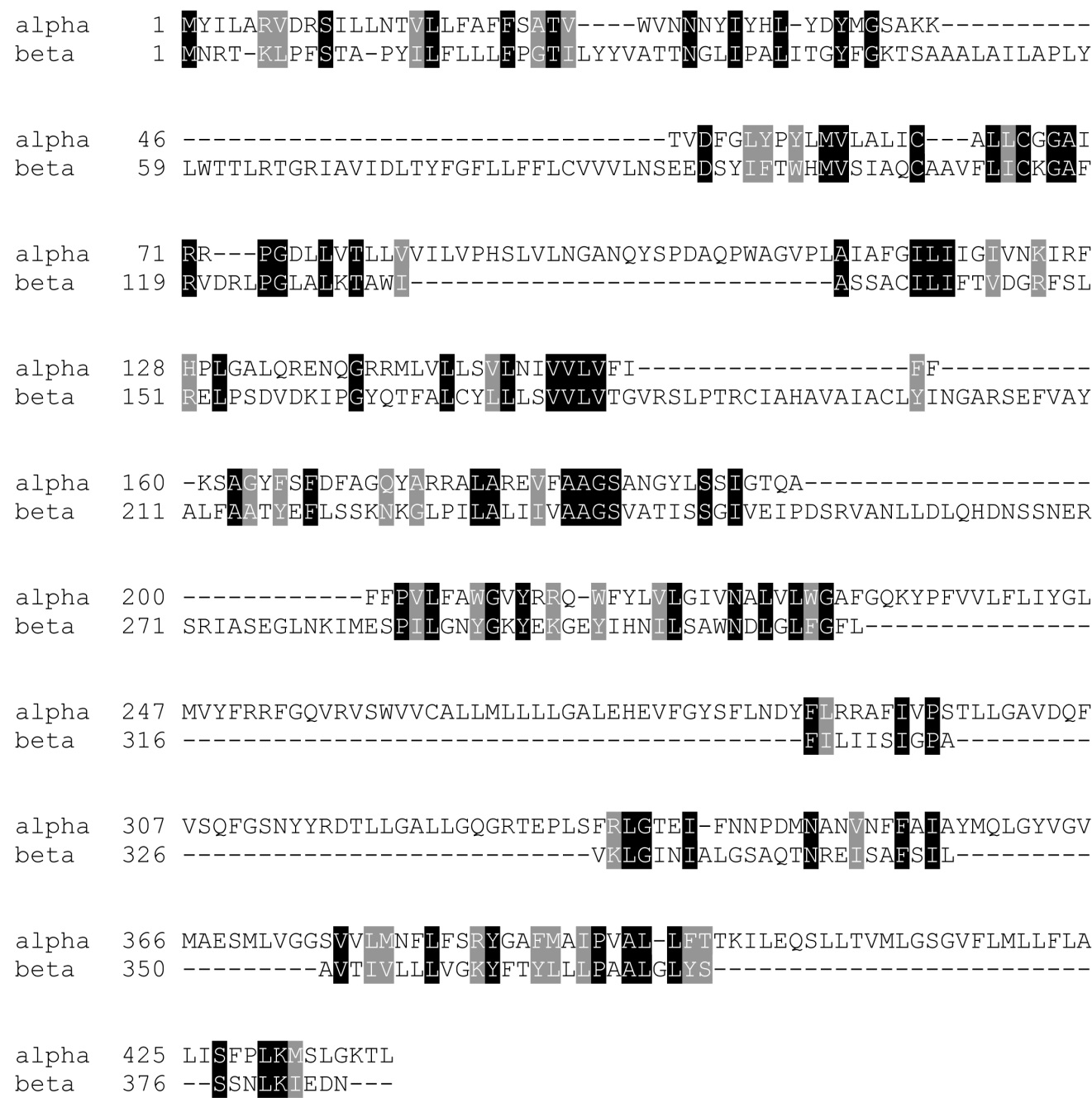
**

**Figure S2 *De novo* topology of Wzy_β_. The resulting output was generated by HMMTOP 2.0 and TOPCONS generated by the Protter server (http://wlab.ethz.ch/protter/start/) (**[**Omasits*, et al.*, 2014**](#_ENREF_186)**).**

**
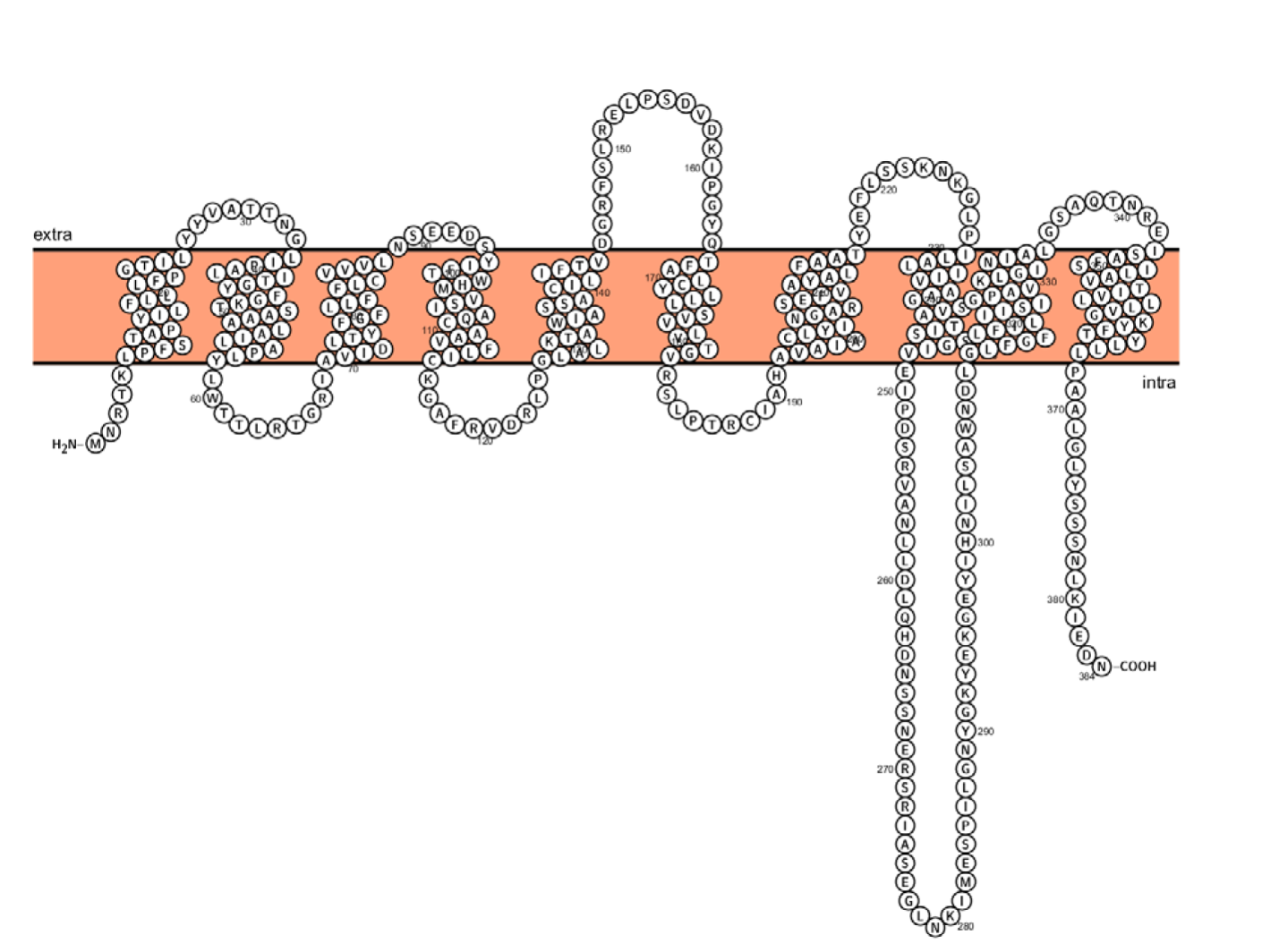
**

# Figure S3: Membrane localization of Wzy_β_ mutants in a T363 background. Site-directed mutagenesis of essential residues was performed in a pPLEO1-T363 background and grown on dual-indicator plates in order to confirm membrane localization: i) pPLEO1-empty ii) pPLEO1-Wzy_β_ iii) pPEO1-T363 iv) R147A-151A v) R254A vi) R270A vii) R272A viii) K295A and ix) H300A.

#
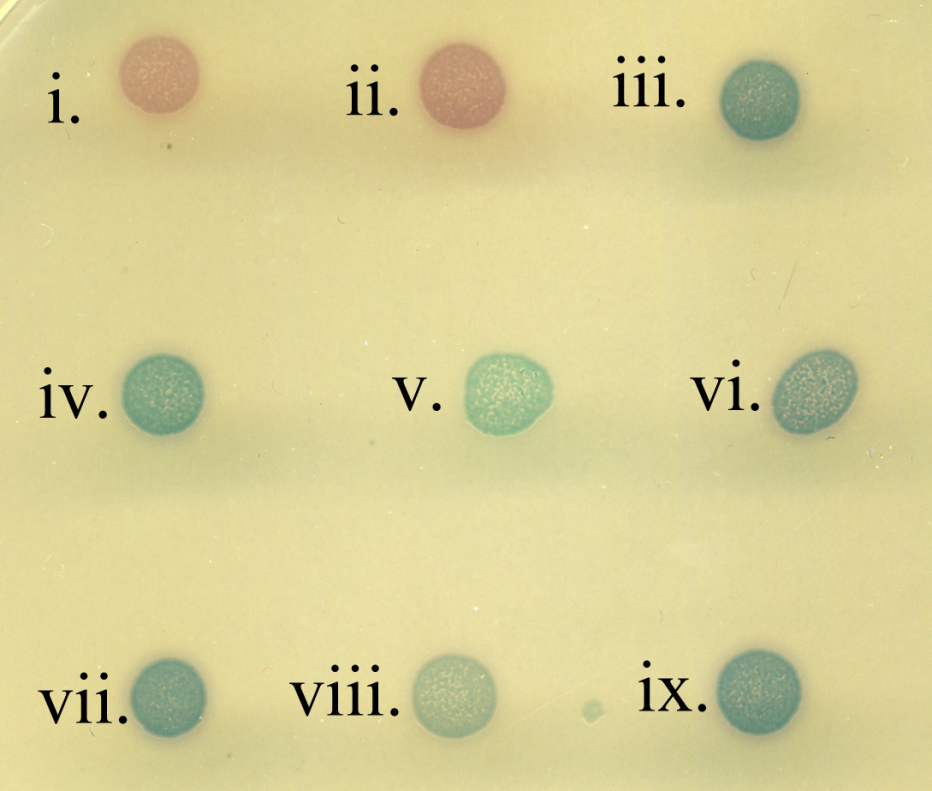


**Figure S4: Western immunoblot of Wzy_β_ and Wzy_α_ in a *P. aeruginosa* serotype O9 background probed with MAb specific to O9 (MF43-3) and inner core (5c7-4).**


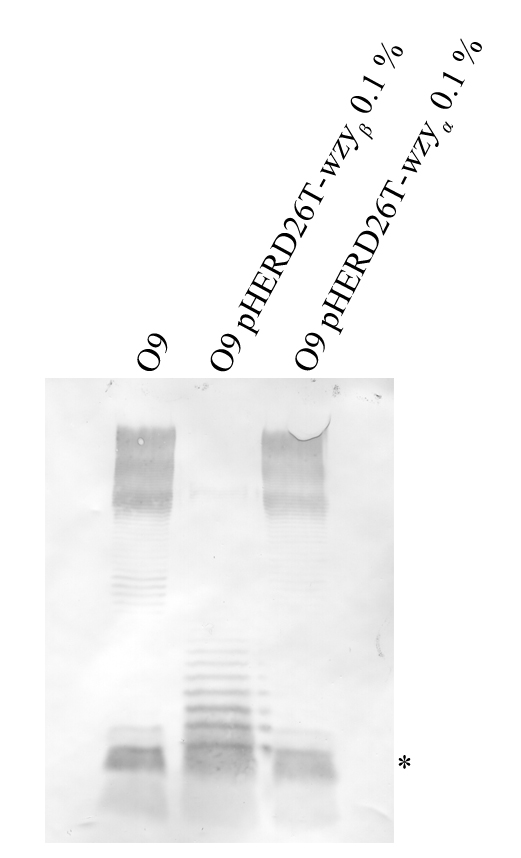


# Table S1: Oligonucleiotide primers

| **Name** | **Sequence 5’ to 3’** |
| --- | --- |
| pPLEO1*wzy_β__*F | ctagcatggagctcatgaataggaccaagcttccg |
| pPLEO1*wzy_β_ _*R | agtcagtctctagaaattatcctcgattttagattgg |
| *wzy_β_*G68*_*R | catgcatgctgcagtattcgtcctgttctgagcg |
| *wzy_β_*D121 *_*R | catgcatgctgcagaagagctagcccaggtagtcg |
| *wzy_β_*G180*_R* | catgcatgctgcagtcccgtaacaagaacaaccg |
| *wzy_β_*L220*_*R | catgcatgctgcagaagtatagggagccccttgt |
| *wzy_β_*L230*_*R | catgcatgctgcagaagtatagggagccccttgt |
| *wzy_β_*L318*_*R | catgcatgctgcaggccgaatagtcctaggtcattc |
| pHERD26T*wzy_β__*F | ctagcatggagctcatgaataggaccaagtcttccg |
| pHERD26T*wzy_β__*R | agtcagtcggatccctaaaatcgaggataattaactgcagg |
| *wzy_β_*R147Asense | tagacggtgcattctcgcttag |
| *wzy_β_*R147Aantisense | ctaagcgagaatgcaccgtcta |
| *wzy_β_*R151Asense | agattctcgcttgcagaactcccagc |
| *wzy_β_*R151Aantisense | gctggggagttctgcaagcgagaatct |
| *wzy_β_*R147A-151Asense | gcattctcgcttgcagaactccccagc |
| *wzy_β_*R147A-151antisense | gctggggagttctgcaagcgagaatgc |
| *wzy_β_*R182Asense | gggagtggcatcactgcc |
| *wzy_β_*R182Aantisense | ggcagtgatgccactccc |
| *wzy_β_*R254Asense | cagatttgctactgcactatctgg |
| *wzy_β_*R254Aantisense | cagatttcgtactgccactatctgg |
| *wzy_β_*R270Asense | caagcaatgaggcgagccggatagc |
| *wzy_β_*R270Aantisense | gctatccggctcgcctcattgcttg |
| *wzy_β_*R270Ksense | caatgagaagagccggatagcatc |
| *wzy_β_*R270Kantisense | gatgctatccggctcttctcattg |
| *wzy_β_*R272Asense | atgagcggagcgcgatagcatctgaag |
| *wzy_β_*R272Aantisense | cttcagatgctatcgcgctccgctcat |
| *wzy_β_*R272Ksense | gcggagcaagatagcatctgaag |
| *wzy_β_*R272Kantisense | cttcagatgctatcttgctccgctc |
| *wzy_β_*K292Asense | actatggagcatatgaaaaaggcg |
| *wzy_β_*K292Aantisense | cgcctttttcatatgctccatagt |
| *wzy_β_*K295Asense | gaaaatatgaagcaggcgagtacatac |
| *wzy_β_*K295Aantisense | gtatgtactcgcctgcttcatattttc |
| *wzy_β_*H300A | aaggcgagtacatagccaacatcctttcagcatg |
| *wzy_β_*H300Aantisense | catgctgaaaggatgttggctatgtactcgcctt |
| *wzy_β_*H300Rsense | cgagtacatacgcaacatcctttc |
| *wzy_β_*H300Rantisense | gaaaggatgttgcgtatgtactcg |
